# Metabolic and immune/inflammatory alterations induced by a triathlon under extreme conditions

**DOI:** 10.3389/fspor.2022.915343

**Published:** 2022-08-17

**Authors:** Cesar Miguel Momesso Santos, Jônatas Bussador Amaral, Marcelo Rossi, Rodolfo Paula Vieria, Cesar Cavinato Cal Abad, André Luis Lacerda Bachi

**Affiliations:** ^1^Interdisciplinary Post-Graduate Program in Health Sciences, Cruzeiro Do Sul University, São Paulo, Brazil; ^2^Faculty ENAU, Ribeirão Pires, SP, Brazil; ^3^ENT Lab, Department of Otorhinolaryngology-Head and Neck Surgery, Federal University of São Paulo (UNIFESP), São Paulo, SP, Brazil; ^4^Post-graduation Program in Human Movement and Rehabilitation and in Pharmaceutical Sciences, Evangelical University of Goias (Unievangélica), Anápolis, GO, Brazil; ^5^Brazilian Institute of Teaching and Research in Pulmonary and Exercise Immunology (IBEPIPE), São José dos Campos, SP, Brazil; ^6^Post-Graduation Program in Bioengineering, Universidade Brasil, São Paulo, SP, Brazil; ^7^Post-Graduation Program in Sciences of Human Movement and Rehabilitation, Federal University of São Paulo, Santos, SP, Brazil; ^8^Reference Center of Sport Science - Social Service of Industry (SESI), São Paulo, SP, Brazil; ^9^Faculty Lusófona, Cotia, Brazil; ^10^Post-Graduation Program in Health Sciences, Santo Amaro University (UNISA), São Paulo, SP, Brazil

**Keywords:** exercise physiology, exercise immunology, cytokines, strenuous exercise, cycling

## Abstract

**Purpose:**

To investigate the effects of triathlon racing under extreme conditions on metabolic and immune/inflammatory responses.

**Methods:**

Thirteen amateur athletes participated in an extreme triathlon competition (swim – 3.8 km; cycling – 180 km; running – 4 2 km; with a 3,700 m accumulated altitude). Blood samples were collected on three different occasions: pre-competition (baseline), immediately post-competition (IM), and 12 h post-competition (12 h) to evaluate glycemic and lipid profiles, leukocytes count, and cytokines levels in plasma and in whole-blood cell culture supernatant stimulated or not with LPS.

**Results:**

Decreased glucose and triglycerides levels, increased LDL, and a significant leukocytosis were observed at IM and 12 h compared to baseline. In addition, higher serum levels of IL-6, IL-8, and IL-10 were found at IM than in baseline and post-12 h. Whereas increased IL-12p40 levels were observed for 12 h compared to baseline. At baseline, in LPS-stimulated cell culture, IL-6, IL-8, and IL-12p70 were higher, while IL-12p40 levels were lower than non-stimulated cell culture. At IM, IL-12p40 levels were unchanged, while higher levels of other cytokines were found in LPS-stimulated cell culture compared to non-stimulated cell culture. The 12 h results showed higher levels of IL-6, IL-8, and IL-10 in LPS-stimulated cell culture than in non-stimulated cell culture. Additionally, a significant negative correlation between circulating glucose levels and IL-6 was found.

**Conclusion:**

The triathlon competition's performance under extreme conditions has remarkable impacts on the lipid profile and systemic immune/inflammatory responses. For the first time, significant alterations in the cytokine responses of whole blood cell culture to LPS-stimulation in baseline, IM, and 12h were demonstrated.

## Introduction

According to the World Health Organization (Bull et al., 2020), to achieve benefits in health, it is necessary to accumulate 75–150 min per week of high- to moderate-intensity physical activity. Moreover, it has been reported that short to middle distance/duration exercise training performed in high intensity, as well as long to very long exercise training (i.e., >2 h) performed in low-to-moderate intensity can decrease the immune response and, consequently, increase the incidence of upper respiratory infections (Vaisberg et al., 2013; Keaney et al., 2018; Simpson et al., 2020). Similarly, it is important to highlight that, more recently, the literature is considerating that other conditions, such as anxiety, interruption of sleep, travel, exposure, nutritional deficits, and environmental extremes, can also impact the immune disturbance frequently observed in athletes (Suzuki et al., 2006; Walsh et al., 2011), mainly when the practitioners are exposed to an overload training that exceeds the physiological personal limits, a situation known as overreaching and/or overtraining (Meeusen et al., 2013).

It was documented that, after a long-term endurance exercise session, such as a marathon or an ultra-triathlon race, there is not only physiological and biochemical impairment but also immunological impairment (Nieman et al., 2004; Suzuki et al., 2006). In fact, a prominent leukocytosis and significant alteration in the immune function are observed after a prolonged and strenuous exercise session (Baum et al., 1997). Beyond these features, Nieman et al. (2004), 1.5 h after an Ironman run, found an increase in the plasma concentration of interleukin (IL-6, IL-8, and IL-10) and interleukin-1 (IL-1ra) antagonist receptors, whereas Jeukendrup et al. (2000), reported that serum levels of tumor necrosis factor-alpha (TNF-alpha) were unchanged both during and after long-distance triathlon. Overall, these acute increases in cytokine responses signal an immune compromise with a concomitant increase in the risk of infections after long-lasting events (Nieman, 1994).

Interestingly, it has been pointed out that the exercise-related cytokine response is not only different from infections but also presented a fascinating cascade that can influence the control of inflammatory response and the regulation of some crucial metabolites necessary to exercise performance. In fact, the IL-6 release by muscle contraction and its detection in the circulation precedes the appearance of other cytokines. In this sense, it is known that IL-6 released in the exercise context precedes the release of IL-1ra, IL-10, and the TNF-alpha soluble receptor, favoring an anti-inflammatory modulation to avoid the exacerbation of inflammation induced by the exhaustive exercise (Walsh et al., 2011).

Beyond this action, IL-6 shows a remarkable metabolic effect, since the higher IL-6 level in response to exercise has been closely related to muscle glycogen depletion and since IL-6 acts in the adipose tissue-promoting lipolysis and also in the hepatic tissue-stimulating glucose releasing. This response occurs in order to increase the bioavailability and mobilization of energy substrates to the skeletal muscles (Pedersen and Hoffman-Goetz, 2000). Starkie et al. (2001) described a positive correlation between IL-6 and creatine kinase (CK), suggesting a relationship between muscle injury and its release (Walsh et al., 2011).

Suzuki et al. (2006) reported no data regarding the modulation of cytokines IL-1b, IL-2, IL-4, interferon gamma, and IL-12p40 after ultra-endurance exercises, since these cytokines have a central role in the balance of the immune response (Th1 and Th2 lymphocytes) (Suzuki et al., 2002, 2003). Among the cytokines produced during a strenuous prolonged ultra-endurance exercise (i.e., long-distance triathlon), IL-6 has been the most extensively evaluated. The IL-6 produced by the contracting muscles is ordered as a myokine and has been recognized as a good biomarker for metabolism and the immune system (Ostrowski et al., 2000; Pedersen and Febbraio, 2008).

Concerning the metabolic effects of the IL-6, the higher IL-6 level in response to exercise has been closely related to muscle glycogen depletion since IL-6 acts in the adipose tissue-promoting lipolysis and also in the hepatic tissue-stimulating glucose releasing. This response occurs in order to increase the bioavailability and mobilization of energy substrates to the skeletal muscles (Pedersen and Hoffman-Goetz, 2000). Starkie et al. (2001) described a positive correlation between IL-6 and creatine kinase (CK), suggesting a relationship between muscle injury and its release. About the IL-6 action on the immune system, it has been well demonstrated that it leads to the release of IL-1ra, IL-10, and the TNF-alpha soluble receptor, favoring an anti-inflammatory modulation to avoid the exacerbation of inflammation induced by the exhaustive exercise (Walsh et al., 2011).

Based on the International Triathlon Union (ITU), long-distance triathlon races are characterized according to the distance for each discipline involved (i.e., swimming > 3 km, cycling > 90 km, and running > 20 km). Particularly in the Ironman, the partial distances comprise swimming 3.8 km, cycling 180 km, and running 42 km. Besides the long distances (>200 km), some events have added unfavorable environmental conditions, providing new challenges for the participants. This type of competition has been named extreme ultra-triathlon and its popularity has been increasing exponentially in the last two decades. Otherwise, the studies about extreme ultra-triathlon remain relatively scarce and the fewer ones are limited since they focused only on performance and anthropometric variables (Lucas et al., 2016). In this respect, it has been highlighted that environmental extreme conditions are considered a remarkable stressful agent that imposes significant physiological and immunological alterations in athletes (Shephard, 1998; Keaney et al., 2018).

Thus, it can be perceived that there is a gap to be explored, such as studies that have more elaborate forms of analysis that allow us to observe how the immune system is modulated in the face of exposure to a challenge. It is widely known that stimulation by LPS, an endotoxin present in the cell wall of gram-negative bacteria, which is recognized by the toll-like receptor (TLR)-4 in the cell membrane of mammals, is capable of inducing a rapid response in leukocytes, leading them to the production of various types of cytokines. Based on this ability, the use of LPS in *ex vivo* assays, mainly in cell culture, allows investigating whether stimulated or non-stimulated cells can induce a response to stimulation with LPS. As physical exercise can directly impact the immune response, the use of LPS becomes an interesting strategy not only to assess whether leukocytes are responsive or not but also to compare the pattern of cytokine response in cell culture assays before and after an exercise session.

According to the literature, differences in cytokine profiles can be verified in plasma and cell cultures obtained in athletes both before, during, and after an exercise session ends (Bernecker et al., 2013; Wang et al., 2013; Bachi et al., 2015). Corroborating this information, we were able to demonstrate that the cytokine profiles observed in immune cell culture from marathon runners showed differences from those verified in the serum at the same time points assessed (Bachi et al., 2015). Thus, in order to better understand the immune/inflammatory effect of an extreme exercise session, it could be appropriated to perform both plasma/serum and cell culture analysis.

To date, there is little information about the inflammatory or immune responses after extreme ultra-endurance exercises. This knowledge may be important since it could help to comprehend better biological responses after long-term exercises are performed under extreme conditions. Therefore, owing to the lack of information concerning the effect of triathlon under extreme conditions on metabolic, inflammatory, and immune responses, the present study was designed aiming to investigate the leucocytes count and the cytokines levels in systemic and cell culture as well as the glycemic and lipid profiles before, 5–10 min post, and 12 h post an extreme triathlon racing.

## Methods

### Subjects of the study and experimental design

**Figure 1:**
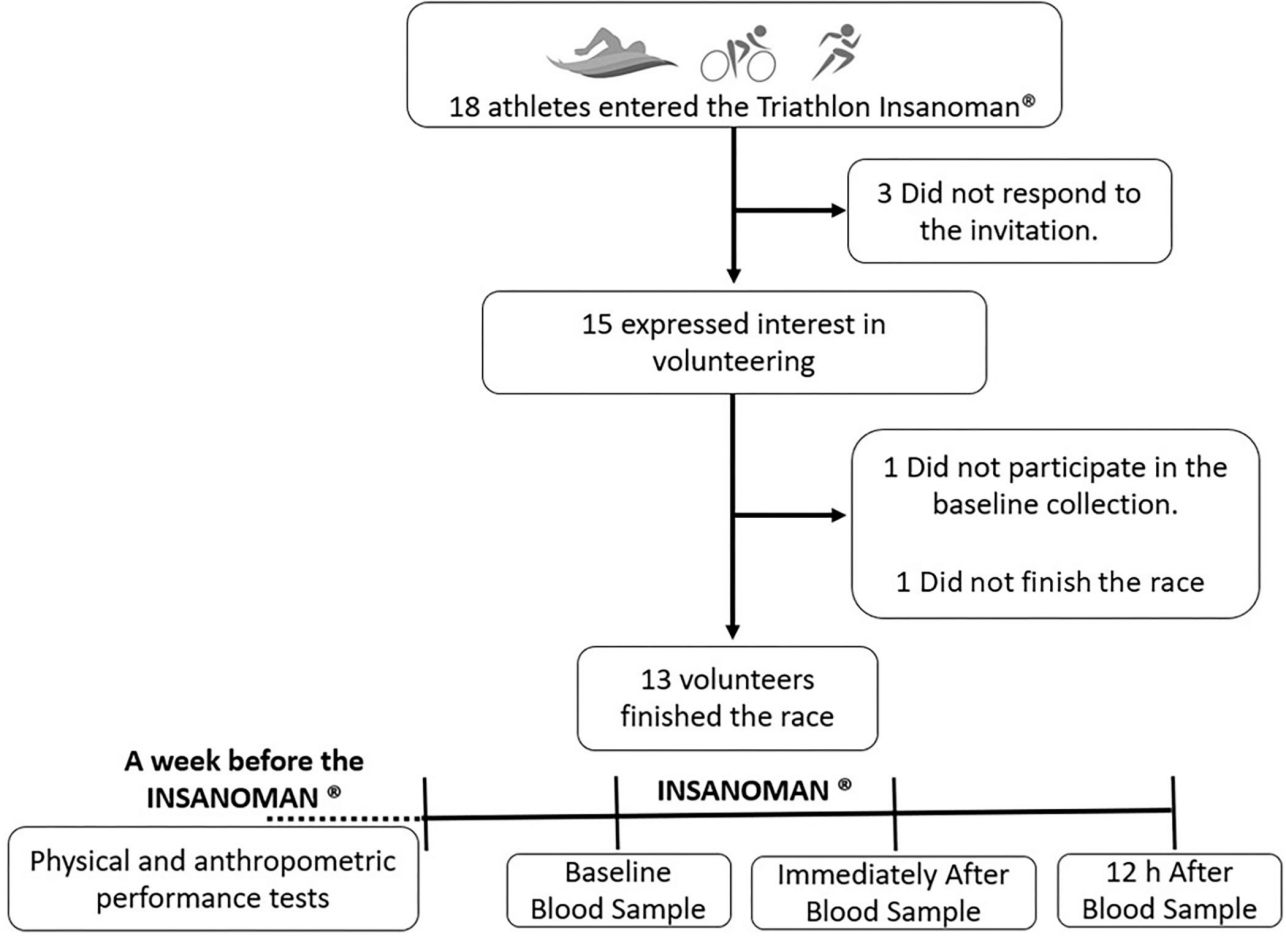
Flow diagram and experimental design of the study.

In the present study, as shown in the flow diagram, 13 amateur athletes 37.00 ± 6.00 years (age), 77.25 ± 2.82 kg (weight), 176.29 ± 5.96 cm (height), 21.84 ± 2.25 (kg.m^2^) (BMI), and 11.08 ± 4.07 % body fat (fat pecentage) participated in all time points of the study's experimental design (Figure 1). In this respect, the experimental design (Figure 1) of the study consisted of three different time-points: (1) from 4 to 7 days before the triathlon extreme competition, the volunteers submitted to the clinical and physical evaluations, emphasizing the ergospirometry tests both performed in the bicycle and in the treadmill. At the same time point, the first blood sample was collected. (2) Immediately after the competition, the second blood sample was collected. (3) 12 h after the end of the competition, the third blood sample was collected (Figure 1). The volunteers participated in a triathlon extreme competition named “INSANOMAN^®^” in 2018.

All volunteers signed the informed consent form previously approved by the National Research Ethics Committee (CAAE number: 39658514.8.0000.5493) and by the local Research Ethics Committee (number: 3,705.954). It is important to cite that the study was performed both in accordance with the Ethical Standards presented in 2016 by Harris and Atkinson (Harriss and Atkinson, 2009) and in accordance with the Declaration of Helsinki (World Medical Association Inc, 2009).

### Triathlon extreme competition – INSANOMAN^^®^^

The triathlon extreme competition, named INSANOMAN^®^, was held in São Paulo, Brazil. Similar to the classic triathlon extreme competitions, the INSANOMAN^®^ consisted of 3.8 km of the swimming, 180 km of cycling, and 42 km of running, but in this competition, a total of 3,700 m of accumulated altitude was also included. The swim stage began in the Igaratá city, at 5:00 am, with the water temperature at 12 °C and altitude at 745 m. The cycling stage concluded at 1,628 m above sea level. It is noteworthy to clarify that the bike route contained a 12.7 km uphill with an average slope of 5.6% and, at the top of “Serra do Paiol,” a 7 km uphill with steep curves and an average slope of 13.4% to the point higher. The temperature at the end of the cycling stage was 25 °C. During the stage of the run, after a flat part, the athletes ran 1.5 km uphill with 500 m of altitude gain. In addition, the temperature at the end of this stage was 5 °C.

### Ergospirometry test

In order to characterize the sample and verify the health status in the face of maximum efforts, the volunteers performed an ergospirometry test on the bicycle and on the treadmill where the cardiometabolic and mechanical variables were obtained according to the protocol used in previous studies (Bentley et al., 2003; Muñoz et al., 2014).

The maximum cycling test was performed on the athlete's own bicycle coupled with a cycle simulator (I-GENIUS MULTIPLAYER; TACX^®^, Netherlands). It measured power and revolutions per minute (rpm). After 8 min of warm-up (two with free power and cadence and six at 150 W and cadence between 80 and 90 rpm), the test started at 150 W, with increments of 25 W every minute until exhaustion. The cadence was maintained between 80 and 90 rpm and the gear ratio was fixed throughout the test. The test was interrupted when the athlete was unable to sustain the desired cadence or voluntary exhaustion.

After 15 min, the maximum incremental run test was started. It was driven on a treadmill (h/p/ Cosmos, HP-Cosmos Sports & Medical GmbH, Nussdorf-Traunstein, Germany) and started at 8 km.h^−1^. The slope was constant (1%) during the test. The treadmill speed was increased by 1 km.h^−1^ every minute until voluntary exhaustion.

During the two tests, the oxygen consumed (VO_2_) was measured breath by breath using a gas analyzer (METALYZER 3B, Cortex^®^, Germany) and averaged at 30-s intervals. Heart rate (HR) was assessed during the test with an HR monitor (RS800CX, Polar^®^, Finland).

In terms of the first ventilatory threshold (VT1) determination, it was achieved when the increase of equivalent of oxygen (VE/VO_2_) was observed along with the oxygen partial pressure at the end of expiration (PETO_2_), concomitantly with the first break in ventilation linearity. In terms of the second ventilatory threshold (VT2), it was achieved when the lowest value of CO_2_ equivalent (VE / VCO_2_) and highest pressure of CO_2_ at the end of expiration (PETCO_2_) were observed, concomitantly with the second break linearity in increasing ventilation (Meyer et al., 2005).

The VO_2_ peak was applied when, at least, two of the following parameters were observed: (1) respiratory exchange ratio > 1.10, (2) heart rate value ≥ 90% of the maximum frequency predetermined by age, or (3) the participant's inability to keep under pressure by verbal encouragement. The highest speed achieved during the test was recorded as the peak speed.

### Collection of the samples

Peripheral blood samples were collected in tubes containing the anticoagulant EDTA on three different occasions: baseline, which corresponds to 4 to 7 days before the competition, with the last exercise training session performed 48 h at rest, immediately after, and 12 h after the competition ends. The blood samples used to perform blood cell culture were kept cold until their use in the assays. It is noteworthy to clarify that the blood sample collection did not occur in the fasting context and that a limitation of this study was the lack of information regarding the food consumption record.

After blood sampling collection, one tube was centrifuged at 900x g for 10 min at 4 °C. A minimum of 500 μL of plasma was stored at −80 °C for later use to evaluate the lipid profile, circulating glucose levels, and cytokine concentration.

### Leukocytes counting

The leukocytes' counting (in percentage) was performed using a semi-automated system (Coulter AcTDiff model, Beckman Coulter, USA). Plasma volume was estimated according to the described by Strauss and collaborators (Strauss et al., 1951), on the basis of hematocrit (%) and hemoglobin (g/dL) results found in the three different time-points assessed in this study.

### Blood cell culture

A volume of 100 μl of the whole blood sample was mixed with 900 μl of RPMI 1,640 containing bovine fetal serum (10%) directly in 48-well plates. Immediately after, the whole blood cell culture was stimulated with lipopolysaccharide (LPS) at a concentration of 10 ug/ml. As a control, another whole blood cell culture was not submitted to LPS-stimulation. Then, the whole blood cell cultures, stimulated or non-stimulated, were maintained at 37 °C in a humidified atmosphere with 5% of CO_2_ for 48 h. After this time, the supernatant of these cultures was collected and submitted to a round of centrifugation (at 900x g for 5 min) in order to remove any cells. Lastly, the cell-free supernatant was frozen at −80 °C for later use to determine the cytokine concentration. The stimulation by LPS promotes a challenge for the leukocytes, stimulating the production of cytokines, allowing to observe the functional capacity of the immune system of an individual to defend itself against invading microorganisms. With physical exercise directly influencing the immune response, it becomes an interesting strategy to compare the cytokine response pattern *in vitro* before and after exercise.

### Determination of plasma cytokines concentration

Cytokine concentrations of IL-2, IL-6, IL-8, IL-10, IL-12p40, and IL-12p70, both in plasma and in the whole blood cell culture supernatant stimulated or non-stimulated with LPS, were determined by ELISA kits (R&D System, Minneapolis, MN, USA), following the manufacturer's instructions. All the cytokines' concentrations were calculated using appropriate standard curves and, in all of them, correlation coefficients from 0.95 to 0.99, with coefficients of variance intra-assay varying from 3 to 5% and from 8 to 10% in inter-assay, were found.

## Statistical analysis

Initially, the sample size was calculated based on IL-6 due to the fact that this molecule is the most evaluated cytokine/myokine in studies involving a strenuous and prolonged exercise session, as reported by Alves et al. (2022). In addition, we emphasize that we use the G^*^Power software and the data obtained in the study developed by Nieman and collaborators (Nieman et al., 2005), and based on a maximum permissible error margin of 5% and a sampling power of 0.95, the minimum sample size calculated were 10 subjects.

The Shapiro–Wilks test was performed to check the normality hypothesis. Descriptive statistics from anthropometrics and biochemical variables were made by mean and standard deviation. Comparisons of parametric (or non-parametric) aforementioned variables were performed, respectively, by ANOVA and the Bonferroni *post-hoc* tests or the Wilcoxon signed-rank test (as appropriated), to verify the differences among baseline, post, and 12 h post-extreme ultra-endurance training. Force of association from the performance of participants in competition was obtained by the Pearson's product–moment correlation coefficient. All statistical analyses were performed by GraphPad Prism 9.0.0. The level of significance was a *p* ≤ 0.05.

## Results

Table 1 shows the metabolic parameters and the results related to the performance in the competition (triathlon - INSANOMAN) of the athlete participants in the study.

**Table 1:** Data related to anthropometric, metabolic, and performance characteristics of the athlete volunteers in the study.

**Variables**	**Mean (±SD)**
Cycling test	
VT1 (ml.kg.min^−1^)	37.80 (± 4.60)
VT1 (%VO_2_max)	70.20 (± 4.10)
VT2 (ml.kg.min^−1^)	47.50 (± 6.30)
VT2 (%VO_2_max)	88.00 (± 4.70)
VO_2_max (ml.kg.min^−1^)	53.91 (± 7.87)
Maximum load (W)	374.62 (± 59.11
Running test	
VT1 (ml.kg.min^−1^)	44.80 (± 5.10)
VT1 (%VO_2_max)	82.20 (± 7.60)
VT2 (ml.kg.min^−1^)	51.30 (± 7.10)
VT2 (%VO_2_max)	93.50 (± 3.00)
VO_2_max (ml.kg.min^−1^)	54.64 (± 8.48)
Maximum load (km.h^−1^)	17.45 (± 1.52)
Racing performance	
Swimming time (hh:mm:ss)	01:10:55 (± 00:10:05)
Cycling time (hh:mm:ss)	09:29:37 (± 01:14:36)
Running time (hh:mm:ss)	05:05:00 (± 00:56:34)
Total race time (hh:mm:ss)	15:45:32 (± 02:06:44)

VT1, first ventilatory threshold; VT2, second ventilatory threshold; VO_2_max, maximal oxygen uptake.

Table 2 shows the data concerning circulating glucose levels, lipid profile, leukogram, and plasma volume obtained in the three different time points: before (baseline), immediately after the competition ends (post), and 12 h after the competition ends (12 h). It is possible to observe that the circulating glucose levels were significantly decreased at post (*p* = 0.011) and 12 h (*p* = 0.035) time points as compared to the baseline values. In relation to the lipid profile, higher levels of LDL (*p* = 0.021) and lower levels of triglycerides (*p* = 0.017) were found in post and 12 h time points in comparison to the baseline values. No other significant differences were observed in the lipid profile.

**Table 2:** Values [mean and standard deviation (SD) of circulating glucose levels, lipid profile (total cholesterol, cholesterol non-HDL, HDL, LDL, and triglycerides, in mg/dL), leukogram (total leukocytes, lymphocytes, monocytes, and granulocytes, in %), and plasma volume (in %) of volunteers in three different time-points: baseline, IM and 12h post-extreme ultra-triathlon race.

	**Baseline**	**IM**	**12 h**
	**Mean ±SD**	**Mean ±SD**	**Mean ±SD**
Circulating Glucose Levels (mg/dL)	127.4 ± 38.25	98.88*± 18.02	105.20*± 10.56
Lipid profile (mg/dL)			
Total cholesterol	158.9 ± 11.78	165.2 ± 17.56	173.9 ± 21.68
Cholesterol non-HDL	118.1 ± 11.88	122.6 ± 16.74	132.00 ± 20.80
HDL	40.78 ± 1.61	42.61 ± 2.99	41.92 ± 2.03
LDL	66.12 ± 11.90	88.00*± 24.88	89.91*± 21.00
Triglycerides	259.9 ± 77.58	197.8*± 36.01	210.3*± 21.80
Leukogram (%)			
Total leukocytes	6.25 ± 1.45	16.19*± 1.88	10.69*^#^ ± 2.65
Lymphocytes	21.91 ± 0.08	11.79*± 2.90	**21.63**^#^ ± 6.34
Monocytes	12.16 ± 8.55	9.94 ± 2.57	11.31 ± 2.11
Granulocytes	64.01 ± 9.93	77.24*± 3.07	**67.13**^#^ ± 7.22
Plasma volume (%)	100 ±	108.43 ± 20.63	109.59 ± 20.72

* p < 0.05 in relation to baseline values;

# p < 0.05 in relation to IM values. Bold values indicate the statistical differences.

Regarding the leukogram analysis, a significant leukocytosis was observed (*p* < 0.05) at the post time point, which lasted till 12 h, as compared to the baseline values. However, it is important to mention that the values found at 12 h were lower than post-time point. Interestingly, whereas a lower percentage of lymphocytes were found at the post time point, a higher percentage of granulocytes were observed on this same occasion in comparison to the baseline values. In the 12 h time point, the percentage of lymphocytes increases and granulocytes decrease in comparison to the post time point values, returning to the baseline. Lastly, the plasma volume unchanged in the three time points was evaluated.

**Figure 2:**
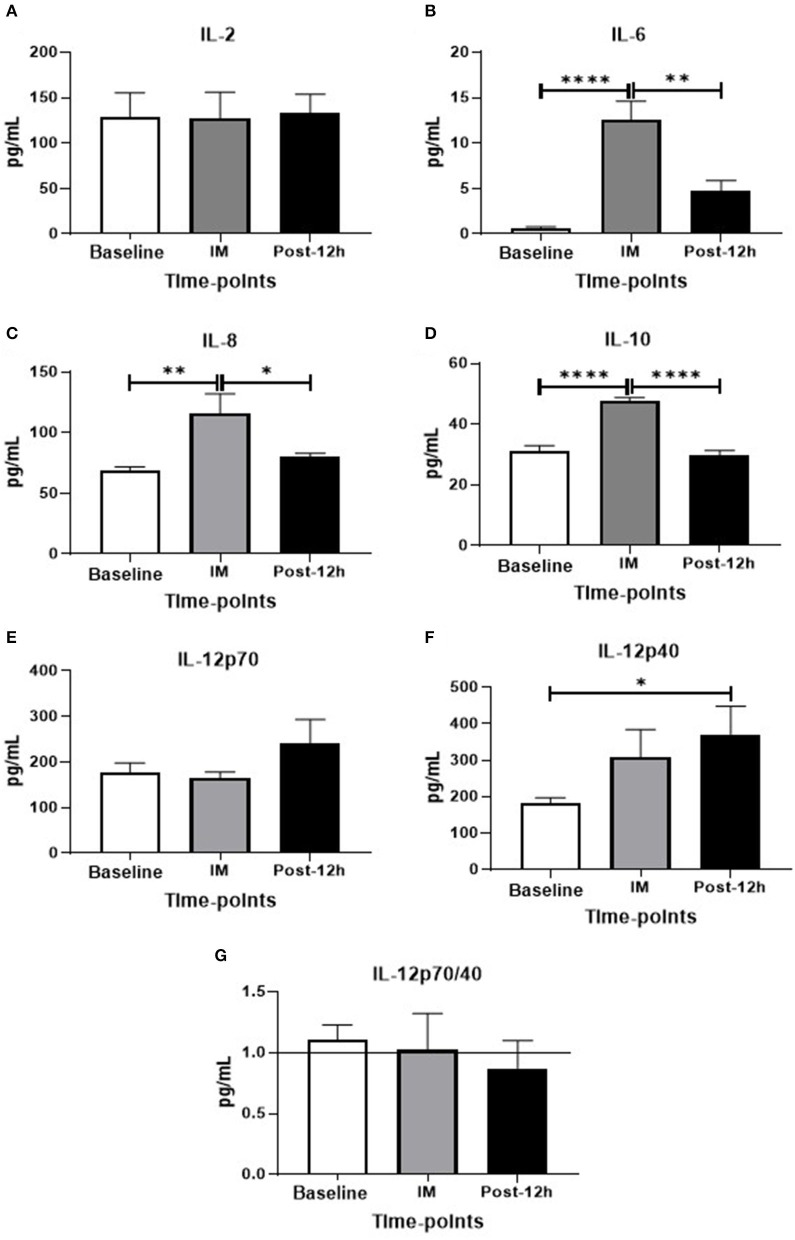
**(A–G)** Levels of plasma cytokines in different time-points: *p < 0.05, **p < 0.01, ***p < 0.001, ****p < 0.0001.

As seen in Figure 2, higher serum levels of IL-6 (Figure 2A), IL-8 (Figure 2B), and IL-10 (Figure 2C) were found post as compared to the values observed at baseline and 12 h, whereas IL-2 (Figure 2D) and IL-12p70 levels (Figure 2E) were unchanged. Particularly in relation to the serum IL-12p40 levels (Figure 2F), increased levels of this cytokine were observed at 12 h as compared to the levels found at baseline. Although this elevation in IL-12p40 levels had reduced the IL-12p70/IL-12p40 ratio by 12 h, no statistical difference was found (Figure 2G).

**Figure 3:**
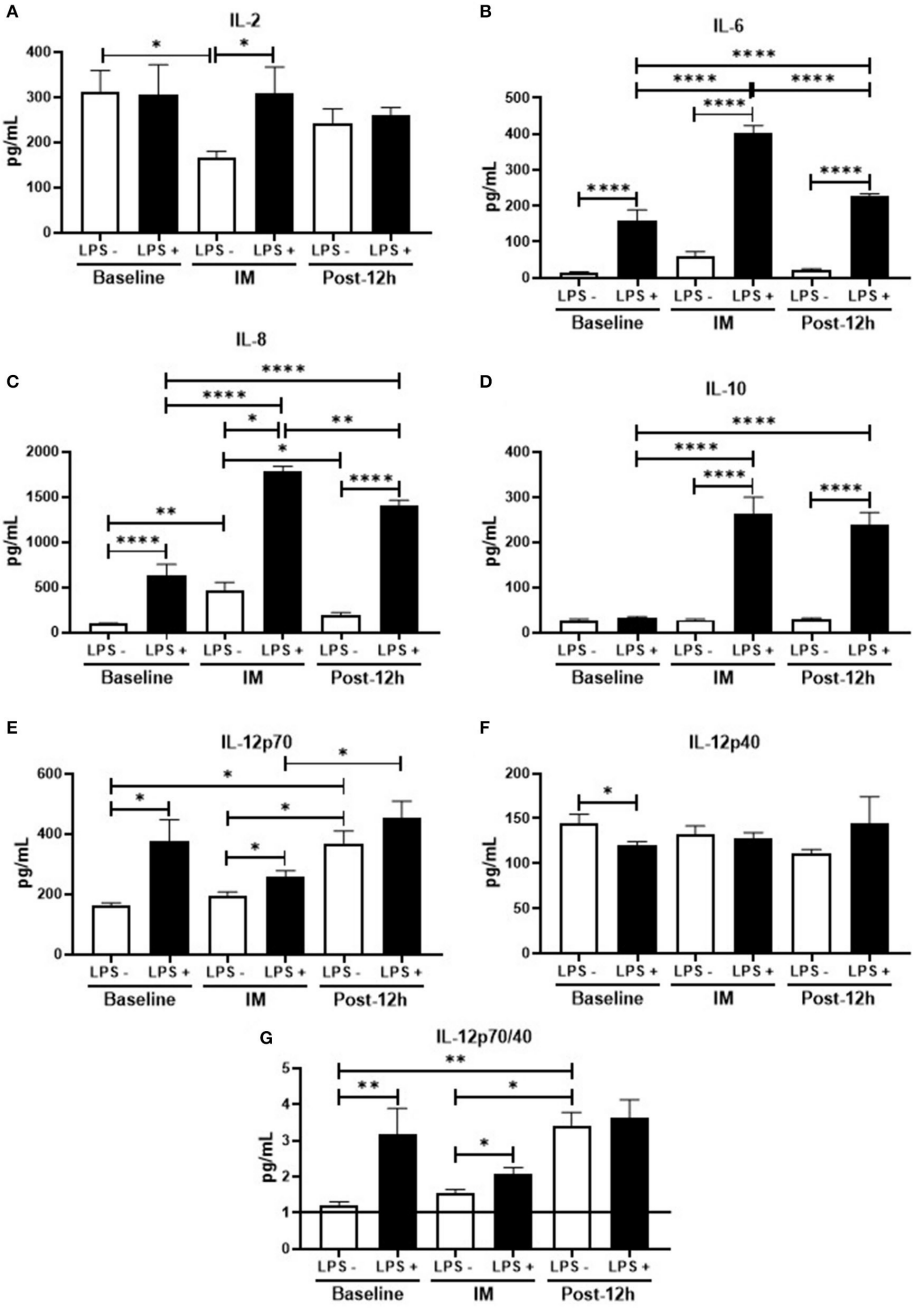
**(A–G)** Profile of inflammatory and non-inflammatory cytokines at the time-points, in LPS^+^ and LPS^−^: *p < 0.05, **p < 0.01, ***p < 0.001, ****p < 0.0001.

Figure 3 shows the results obtained in the cytokines evaluation of peripheral blood mononuclear cell (PBMC) culture. In relation to the data found before, the levels of IL-6 (Figure 3A), IL-8 (Figure 3B), IL-12p70 (Figure 3E), and the IL-12p70/IL-12p40 ratio (Figure 3G) were higher, whereas the IL-12p40 levels (Figure 3F) were lower in stimulated than non-stimulated cell culture. Interestingly, the levels of IL-2 (Figure 3D) and IL-10 (Figure 3C) were unchanged between the stimulated cell culture and non-stimulated cell culture. Concerning the results obtained at post, only IL-12p40 levels (Figure 3F) were unchanged, whereas all the other cytokines showed a significant increase in their levels when stimulated as compared to the non-stimulated cell culture. In addition, it was also observed that not only the levels of IL-2 were lower (Figure 3D), while the IL-8 (Figure 3B) were higher in the non-stimulated cell culture in relation to the values found at baseline and 12 h, but also higher IL-6 (Figure 3A), IL-8 (Figure 3B), and IL-10 (Figure 3C) levels were found in the stimulated cell culture in comparison to the values obtained at baseline. Regarding the results obtained at 12 h, a significant increase of IL-6 (Figure 3A), IL-8 (Figure 3B), and IL-10 (Figure 3C) was observed in the stimulated cell culture as compared to the values obtained in the non-stimulated cell culture, whereas the levels of other cytokines evaluated did not differ. In addition, the higher levels found in these cytokines were also significantly increased when compared to the values obtained before the competition but, particularly in terms of (Figure 3A) and IL-8 (Figure 3B), were also lower than the values observed post. Interestingly, the IL-12p70 levels (Figure 3E) and also the IL-12p70/IL-12p40 ratio (Figure 3G) found in the non-stimulated cell culture at this time-point were significantly increased as compared to the values obtained both in baseline and post, whereas only the IL-12p70 levels (Figure 3E) observed in the stimulated cell culture were higher than the values found at post.

**Figure 4:**
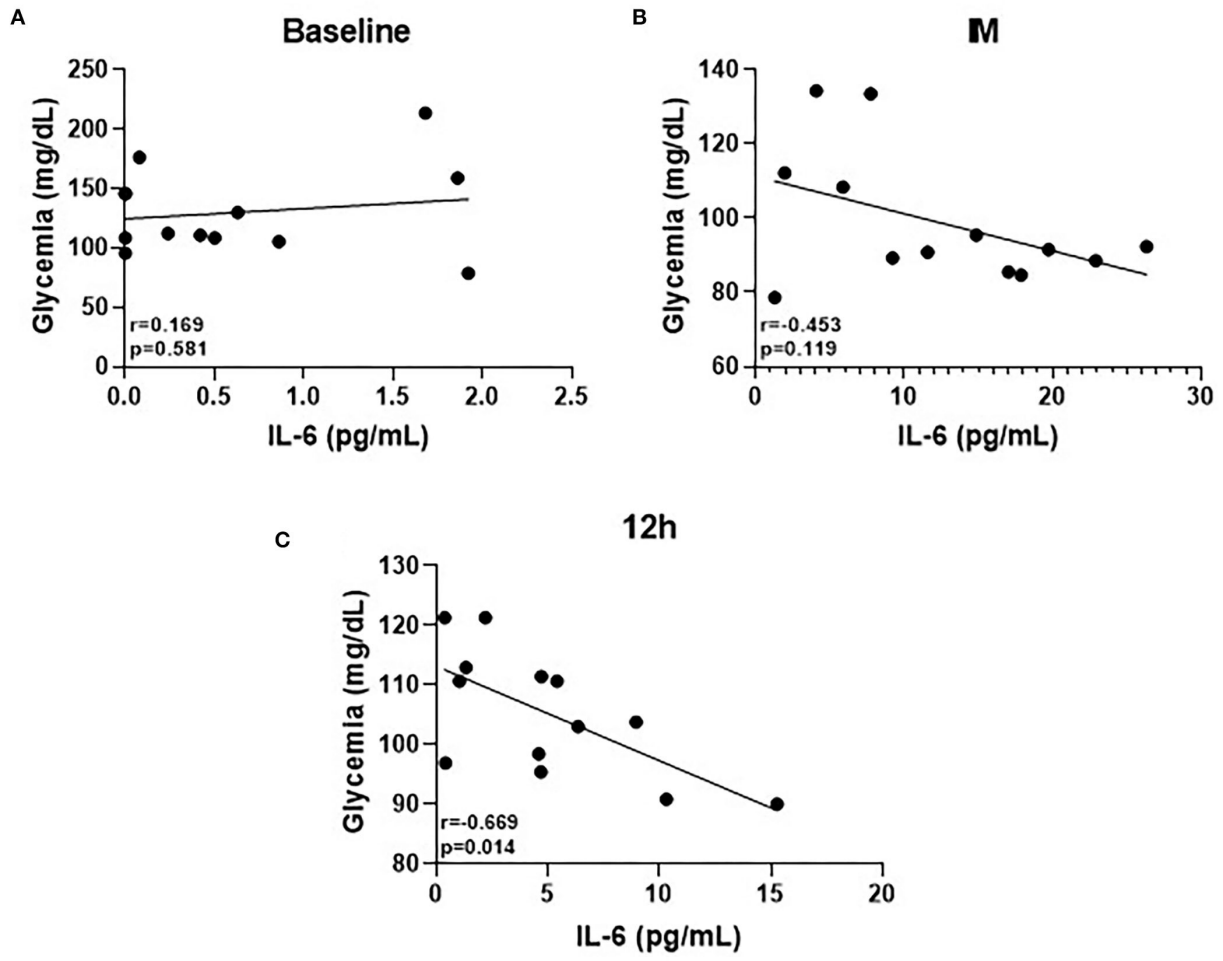
**(A–C)** Pearson's correlation of IL-6 cytokine at the three time-points.

Figure 4 shows the main results obtained in the correlation analysis. No correlation between circulating glucose levels and IL-6 was found both at baseline (Figure 4A) and post time points (Figure 4B). However, at 12 h, a significant negative correlation between these parameters was found (Figure 4C).

## Discussion

The aim of this study was to investigate the leucocyte count and the cytokines levels in both systemic and cell culture as well as the glycemic and lipid profiles before, post, and 12 h post-extreme ultra-triathlon racing. Our findings demonstrated that the performance of triathlon competition under extreme conditions, named “INSANOMAN,” was able to induce significant systemic alteration in the glycemic, LDL, and triglycerides levels as well as in immunological leukocytosis associated with the remarkable elevation in the granulocytes count, besides the increase of pro-inflammatory and anti-inflammatory cytokines. Interestingly, a negative correlation was observed between IL-6 and glycemic levels at 12 h. In addition, among the results obtained in the culture of total blood cells, particularly it was evidenced that the competition not only trigged a higher release of pro-inflammatory cytokines, especially IL-8 and IL-12p70 in comparison to the baseline, as well as in the baseline time points, but also found no differences in the IL-2 and IL-10 levels, regardless of the LPS-stimulation.

Regarding the results showing that the LPS-stimulation did not elicit elevation on IL-10 levels, it is noteworthy to highlight that, in agreement with previous authors (Williams et al., 2004), the lack of IL-10 expression LPS-derived was only observed after 1-h stimulation (Abbasi et al., 2013; Antunes et al., 2019) since it has been described that the IL-10 expression induced by LPS occurs 3–5 h after LPS-stimulation (Jiang et al., 2002). Corroborating this information, Nielsen and colleagues (Nielsen et al., 2016) showed that 6 h of LPS-stimulation was able to induce a significant increase in IL-10 levels in a whole blood cell culture before an exhaustive exercise session. Taking into account these data, our unexpected finding that 24-h LPS-stimulation was not able to elevate IL-10 levels in the cell culture before the competition can be putatively associated with an “anticipatory behavior” related to the competition and/or a modulatory effect of training periodization performed by the athletes participating in this study.

We can consider the hypothesis of “anticipatory behavior” related to competition, due to the fact that it is already widely discussed in the literature that the objective of training periodization is to promote an increase in physical performance, coinciding with specific dates of competition. However, the prescription of physical training, in general, is established by alternating training variables, such as training volume, intensity, or frequency (Muñoz et al., 2014). However, in endurance sports like triathlon, it is common to choose from high-volume training. According to the report by Etxebarria et al. (2019), due to the fact that the triathlon involves three different stages in sequence (swimming, cycling, and running), its training periodization requires careful planning of large amounts of training sessions per week, often more than one session on the same day, which leads to a high level of physical stress.

By the way, it is paramount to mention that, at an appropriate level, the physical stress generated by exercise training sessions are triggers of the physiological adaptation process, promoting improved physical performance. However, when this stress promoted by training is poorly managed, it can result in temporary decreased performance, with high levels of fatigue, commonly associated with increased muscle damage (Sumi et al., 2021), imbalance in immune cells (Cosgrove et al., 2012), modulation of anabolic and catabolic hormones (Urhausen et al., 1995), and mood change (Comotto et al., 2015), which can evolve to overtraining, promoting the loss of performance that can last for months to be reestablished. In order to mitigate this negative status, a strategy often used is, after the period of the intensification of training loads to induce greater levels of adaptation and performance improvement, the athletes use the weeks prior to competition to reduce training loads and enhance supercompensation, a process known as polishing or taper (Clemente-Suárez and Ramos-Campo, 2019). However, Aubry and colleagues (Aubry et al., 2014) demonstrated that, after a period of the intensification of training load, triathletes can develop an overreaching status, with a high perception of fatigue associated with training, compared to athletes who had only acute fatigue during training. Thus, even using the taper as a strategy to achieve supercompensation, other parameters associated with stress, such as mood states, can impact the overreaching development.

In this sense, our group demonstrated that, prior to an exhaustive and prolonged competition, the levels of IL-10 in cell culture without stimulation were higher than, not only, the levels found immediately and 72 h after the competition ends but also the control group. This remarkable finding was associated with increased GH levels, a well-known stress hormone, and also altered mood states, particularly vigor and tension, before the competition, showing that psychological stress arising from anticipating the effort physical can act by modulating several factors involved in the neuro-immune-endocrine context in the athletes prior to the competition (Bachi et al., 2015). Therefore, the lack of elevation of IL-10 levels after LPS-stimulation could be putatively attributed both to the psychological stress elicited by an “anticipatory behavior” related to the eminent challenge and also to the physical training periodization since it was reported that 12 weeks of an exercise training program (including endurance and resistance exercises) was able to induce a significant reduction in the releasing of IL-6 from a whole blood cell culture LPS-stimulated in conjunct with lower TLR4 expression in CD14+ cells (Stewart et al., 2005). Another other possible cause for the levels of IL-10 being unchanged after LPS-stimulation can be associated with the well-known capacity of higher levels of TNF-alpha to block endogenous IL-10 production in monocytes in response to LPS (Rossol et al., 2011). In a general context, in accordance with as reported by Simpson et al. (2020). “The consensus among investigators is that exercise-induced immune changes reflect the physiological and metabolic stress experienced by the individual.”

Corroborating this suggestion of regulatory effect on specific IL-10 expression before the competition, as expected, LPS-stimulation was able to modulate the cytokine expression not only by increasing pro-inflammatory cytokines, such as IL-6, IL-8, and IL-12p70, but also by decreasing IL-12p40, a cytokine with anti-inflammatory properties by its capacity to antagonize the IL-12p70 action (Cordeiro et al., 2021), which led to an elevation of IL-12p70/IL-12p40 ratio.

Regarding the results observed in the whole blood cell culture at the time point immediately after the competition, as an exception to the IL-12p40 expression, the other cytokines evaluated presented a significant increase in their levels 24 h after LPS-stimulation comparing the non-stimulated cell culture. Particularly in terms of IL-10, the increase of this cytokine after LPS-stimulation can support our previous suggestion and demonstrate that a modulatory response was elicited in order to regulate the elevation of pro-inflammatory cytokines found here. Furthermore, the lack of IL-12p40 expression after LPS-stimulation was impacted by the elevation in IL-10 levels since it was reported that higher expression of IL-10 after LPS-stimulation can inhibit the IL-12p40 gene transcription.

Interestingly, the observation that IL-8 levels in the non-stimulated cell culture immediately after the competition was higher than the baseline demonstrates that the competition performance was able to induce *per se* the expression of this cytokine. In agreement with this elevation found in cell culture, a significant increase in serum IL-8 levels was also evidenced at the same time point compared to the baseline results. In terms of systemic IL-8 response to strenuous and prolonged exercise sessions, according to the literature (Chen et al., 2019; Santos et al., 2020), this elevation is expected due to, as formerly cited, IL-8 being a myokine in this context. However, taking into account our finding that IL-8 levels were higher in the whole blood cell culture non-stimulated with LPS allows us to suggest that the serum IL-8 level elevation could be impacted by the secretion both by muscle contraction and also by the circulating cells, especially the immune cells.

Beyond the systemic IL-8 elevation, as also previously mentioned, the observation of a significant increase in the levels of IL-6 and IL-10 immediately after the competition was expected. In a different way of IL-8, the systemic elevation of these two other myokines (IL-6 and IL-10) was only induced by the muscle contraction since no differences in the levels of both these cytokines were observed in the non-stimulated cell culture with LPS immediately after the competition as compared to baseline.

In terms of IL-12 results, although our group demonstrated an increase in the serum IL-12p70 levels immediately after a marathon race (Vaisberg et al., 2019), here, the systemic levels of this cytokine were unchanged as compared to the baseline, which corroborates the literature (Nieman et al., 2001; Sugama et al., 2012). In addition, this lack of elevation in the serum IL-12p70 levels was reflected in the cell culture non-stimulated with LPS since the levels of this cytokine only were different in the IM time-point in relation to the baseline. As expected, the cell culture stimulated with LPS the IL-12p70 levels showed a significant increase. Regarding the results of serum IL-12p40 levels, despite an elevation in their levels, the statistical analysis did not show a significant result. It is utmost to point out that, in the literature, there are a handful of studies showing an increase of this cytokine soon after an exhaustive exercise session (Suzuki et al., 2003, 2006; Sugama et al., 2012, 2013). In relation to the IL-12p40 data obtained in the cell culture, no differences were found when LPS was used at the IM time-point, opposite to that observed in the baseline time point.

Besides these remarkable impacts of triathlon extreme on cytokines, an alteration in the leukocytes profile immediately after the competition was also evidenced. Based on the literature, the significant elevation of leukocytes found here is closely associated with the demarginalization of granulocytes, mainly neutrophils, from the periphery to circulation in response to the catecholamines released during the performance of strenuous and prolonged exercise sessions (Walsh et al., 2011; Foster et al., 2017). So, an increase in the percentage of granulocytes at this time point created an impact, reducing the values concerning the other circulant immune cells, for instance, the lymphocytes percentage, as observed in this study since alterations in the account of lymphocytes after an exhaustive competition does not occur (Mooren et al., 2002; Lancaster et al., 2004). Taken together, the results obtained immediately after the competition ends showed a prominent effect of the triathlon extreme competition in modulating the pattern of immune and inflammatory responses as compared to the baseline.

In order to analyze the repercussions of competition beyond the time point immediately after the competition, we also evaluated another time point after its conclusion (12 h after). First, we were able to report that the percentage of total leukocytes was still raised as compared to the baseline, although the values verified for granulocytes (were higher) and lymphocytes (were lower) presented with only significant differences in relation to the IM values. These observations are in agreement with other studies in which, soon after high-intensity training sessions and/or a strenuous and prolonged exercise session, there was a significant increase of neutrophils (the main granulocytes cells in the blood) in opposite to a decrease of the lymphocytes accounts (Walsh et al., 2011; Morgado et al., 2016). In an interesting way, these altered number of granulocytes and lymphocytes at IM time point could impact our findings at 12 h after the competitions, in which the number of leucocytes was not completely returned to baseline values, as similarly reported in other studies (Lippi et al., 2010; Bartley et al., 2021).

In addition to these observations, as expected, the systemic levels of IL-8, IL-6, and IL-10 were found 12 h after the competition returned to the baseline (Suzuki et al., 2002; Sugama et al., 2013), showing that the release of these myokines by the muscle in response to the performance of triathlon extreme condition was finished in this time point. However, the elevation in the IL-12p40 levels found 12 h after the competition is a very interesting finding, which could indicate that an anti-inflammatory response was elicited in order to regulate the systemic inflammation induced by the competition, although the decrease in the ratio between IL-12p70/IL-12p40 was not significant. To our knowledge, this is the first time that this result is reported since, as formerly cited, an increase in circulating IL-12p40 levels was observed only soon after (immediately or 1–4 h) the competition ends (Suzuki et al., 2003, 2006; Peake et al., 2005; Sugama et al., 2012).

In an interesting way, the higher circulating IL-12p40 levels were not influenced by the increase in its expression on the leukocytes as the levels of this cytokine in cell culture were unchanged at 12 h after the competition, similar to that observed at the IM time point. In opposite, concerning the results of IL-12p70, the whole blood cell culture non-stimulated with LPS showed an elevation of its levels, which demonstrated to us that the performance of the triathlon under extreme conditions was able *per se* to elicit a pro-inflammatory response in these cells, regardless of the LPS-stimulation. It is broadly known that IL-12p70 is one of the major cytokines involved in cellular immunity activation and inflammatory response (Hamza et al., 2010), so the elevation of this cytokine in our study 12 h after the competition allows us to putatively suggest that a cellular immune response was induced in order to contribute with the athlete's recovery. Even though the circulating cells were activated, the systemic release of IL-12p70 was not significantly higher at 12 h after the competition than other time points evaluated here, because, as previously mentioned, a regulatory response mediated by the IL-12p40 was induced. It is worth highlighting that these important findings were not observed for the other cytokines (IL-8, IL-6, and IL-10) since they demonstrated the same expected “behavior” in cell culture at 12 h after the competition as compared to the IM time point.

In accordance with the object of this study, we also proposed to investigate changes in some metabolic parameters, such as blood glucose and lipid profile, induced by triathlon under extreme conditions. In this sense, while circulating LDL-c levels increased, glucose and triglyceride levels were reduced immediately and 12 h after competition compared to baseline. As reported by Lamon-Fava et al. (1989), the acute response to strenuous physical exercise, such as the performance of an endurance triathlon, is accompanied by prominent alterations in the lipid profile, with remarkable reductions in triglyceride levels, especially immediately after the physical exercise session. Corroborating this report, Iurimiaé et al. (1989) also mentioned a reduction in triglyceride concentrations in response to participation in triathlon competitions, and according to the authors, this change could be attributed to the hormonal modulation promoted by endurance triathlon competition. In fact, the pronounced increase in circulating levels of catecholamines and cortisol in association with a moderate increase in somatotropin levels and a decrease in testosterone levels, particularly during strenuous and prolonged physical exercise performance, can induce an increase in the lipolytic activity, leading to an increase in the bioavailability of fatty acids and glycerol, which can be used by muscles to provide energy (Petibois et al., 2003). It is important to mention that the raised exercise-induced lipolytic activity is closely related to an increase in the lipoprotein lipase (LPL)-activity, an enzyme that can elevate the triglycerides levels through the hydrolysis of VLDL and chylomicrons from the circulation (Katsanos, 2006). In this sense, the rapid and increased exercise-induced stimulation of LPL activity aims to supply the cells, particularly muscle tissue, with fatty acids necessary for generating ATP and restoring intracellular triglycerides levels that had been used during exercise sessions (Kersten, 2014). Corroborating these data, Gill and Hardman (Gill and Hardman, 2003) reported that the higher LPL activity upon VLDL during a strenuous and prolonged exercise session diminishes the triglycerides content, consequently driving an increase in the circulating LDL levels. Thus, although we did not assess VLDL level based on these pieces of information, not only an increase in the circulating LDL levels but also reductions in triglycerides levels, as found here, have been evidenced soon after strenuous and prolonged exercise sessions end in response to the increase LPL activity exercise-induced. Consequently, the increment in the bioavailability of triglycerides can offer this fuel for muscle in order to guarantee the energy necessary for the performance of the triathlon under extreme conditions.

As reductions in the levels of triglycerides immediately after a strenuous and prolonged exercise session have been documented in the literature and can indicate that this molecule could be used to provide energy to the athletes performing the competition (Thompson et al., 1980; Kantor et al., 1984), a decrease in glucose levels in the same context is not observed (Anderson et al., 2016). It is noteworthy mentioning that the significant reductions in circulating glucose levels observed both immediately and 12 h after the competition ends were in comparison to the baseline values, in which levels were not obtained in the fasting context. Thus, whether the blood sampling had occurred under fasting conditions, we could putatively suggest that these reductions would be not observed.

Lastly, the significant negative correlation found between glycemia and circulating IL-6 levels 12 h after the competition ends can demonstrate that a metabolic adjustment still persists until this time point. There is no doubt in the literature that the myokine IL-6 has a corollary action as an intracellular metabolic sensor during physical exercise performance (Helge et al., 2003; Reihmane and Dela, 2014), since it has been reported that both the intensity and duration of exercise sessions directly impact the IL-6 release from the muscle, and one of the capacities of this myokine is to improve the fuel provide (both glucose from the liver and fatty acids from adipose tissue) in order to guarantee the performance of exercise session (Febbraio et al., 2004; Reihmane et al., 2013). In fact, a prominent action of IL-6 upon the liver during very demanding exercise has been described, which induces an increase in hepatic glucose production through the upregulation of gluconeogenic gene mRNA levels, particularly phosphoenolpyruvate-carboxykinase (PEPCK) (Banzet et al., 2009). Therefore, the increased circulating IL-6 levels in the athletes who presented lower glucose levels can indicate that this cytokine was released in order to stimulate the hepatic glucose output (Febbraio et al., 2004) and, then, lead to metabolic reestablishment that was not reached in these athletes at this time point.

## Conclusion

In conclusion, we were able to reinforce the prominent effect of strenuous and prolonged exercise sessions in eliciting, acutely, remarkable modulation in some metabolic parameters, especially regarding the circulation levels of LPL, triglycerides, and glucose. Furthermore, for the first time, it is reported in the literature, not only significant alterations in the cytokine responses to LPS-stimulation in the whole blood cell culture before the competition as well as in the immune/inflammatory status both systemic and in cell culture both immediately and 12 h after the competition ended.

## Data availability statement

The raw data supporting the conclusions of this article will be made available by the authors, upon reasonable request.

## Ethics statement

The studies involving human participants were reviewed and approved by National Research Ethics Committee (CAAE number: 39658514.8.0000.5493) and by the local Research Ethics Committee (number: 3,705.954). The patients/participants provided their written informed consent to participate in this study.

## Author contributions

CMMS, RPV, CCCA, and ALLB: research concept and study design. CMMS and ALLB: literature review. CMMS, MR, RPV, JBA, and ALLB: data collection. CCCA and ALLB: data analysis and interpretation. CMMS, MR, RPV, CCCA, and ALLB: statistical analyses. CMMS, JBA, MR, RPV, CCCA, and ALLB: writing of the manuscript, reviewing, and editing draft of the manuscript. All authors contributed to the article and approved the submitted version.

## Conflict of interest

The authors declare that the research was conducted in the absence of any commercial or financial relationships that could be construed as a potential conflict of interest.

## Publisher's note

All claims expressed in this article are solely those of the authors and do not necessarily represent those of their affiliated organizations, or those of the publisher, the editors and the reviewers. Any product that may be evaluated in this article, or claim that may be made by its manufacturer, is not guaranteed or endorsed by the publisher.
